# Efficacy and Safety of Probiotics in Children with Irritable Bowel Syndrome: A Systematic Review and Meta-Analysis

**DOI:** 10.3390/microorganisms14010023

**Published:** 2025-12-21

**Authors:** Marisa Piccirillo, Erika Renzi, Corrado De Vito, Maurizio Mennini, Giacomo Giarrusso, Giorgia Gallo, Giovanna Quatrale, Marco Bianchi, Marco Graziani, Francesca Caron, Alessandro Ferretti, Pasquale Parisi, Giovanni Di Nardo

**Affiliations:** 1 Department of Neurosciences, Mental Health and Sensory Organs (NESMOS), Faculty of Medicine and Psychology, Sapienza University of Rome, 00189 Rome, Italy; marisa.piccirillo@uniroma1.it (M.P.); maurizio.mennini@gmail.com (M.M.); giacomo.giarrusso23@gmail.com (G.G.); giorgia.gallo@uniroma1.it (G.G.); marbi1996@gmail.com (M.B.); m.graziani@uniroma1.it (M.G.); francesca.caron@uniroma1.it (F.C.); alessandro.ferretti@uniroma1.it (A.F.); pasquale.parisi@uniroma1.it (P.P.); 2 Pediatric Unit, Sant’Andrea University Hospital, 00189 Rome, Italy; 3 Department of Public Health and Infectious Diseases, Sapienza University of Rome, 00185 Rome, Italy; erika.renzi@uniroma1.it (E.R.); corrado.devito@uniroma1.it (C.D.V.); 4 Pediatric Gastroenterology and Endoscopy Unit, Department of Pediatric Specialties, Santobono Pausilipon Children’s Hospital, 80129 Naples, Italy

**Keywords:** probiotics, irritable bowel syndrome, children, abdominal pain

## Abstract

Irritable bowel syndrome (IBS) is a disorder of gut-brain interaction (DGBI), whose exact etiology remains unclear. The “brain–gut-microbiota axis” proved to be a key target in IBS management and there is strong evidence supporting the use of probiotics for improving overall symptoms both in adults and in children. We conducted a systematic review and meta-analysis of randomized controlled trials (RCTs) evaluating the effects of probiotic supplementation in pediatric patients diagnosed with IBS according to Rome III or IV criteria. Scopus, PubMed, and Cochrane Library were the available databases systematically searched up to February 2025. Six RCTs with 604 participants were included in the final systematic review. Three RCTs provided data from which the meta-analysis demonstrated that probiotic supplementation has a significant effect on reducing abdominal pain in patients with IBS (SMD −0.95, 95% CI −1.63 to −0.27). Other three RCTs reported data on the effects on stool consistency, and their meta-analysis proved that supplementation results in stool consistency normalization in patients with diarrhea or constipation (OR 2.17, 95% CI 1.18 to 4). The present meta-analysis demonstrated that probiotic supplementation can reduce abdominal pain in pediatric patients with IBS and provide significant bowel habit normalization in patients with diarrhea or constipation at baseline compared to placebo.

## 1. Introduction

Irritable bowel syndrome (IBS) is a disorder of gut–brain interaction (DGBI), defined according to the pediatric Rome IV criteria as recurrent abdominal pain associated with defecation or a change in stool frequency or appearance, occurring at least four times per month for a minimum of two months and not fully explained by another medical condition [1]. IBS is the most prevalent DGBI among children and adolescents [2], with prevalence ranging from 3.6 to 9.9%. The highest prevalence rates have been reported in China, Nigeria and Turkey (13.25%, 16% and 22.6%, respectively) [3,4,5]. In comparison, the lowest rates have been observed in the Mediterranean area and the United States (4% and from 2.8 to 5.1%, respectively) [2]. The considerable variation in reported prevalence may be partly attributed to cultural differences in the interpretation of the Rome criteria, particularly regarding perception of pain and bowel habits. Nevertheless, IBS is widely recognized as having a substantial impact on the daily activities, education, and health-related quality of life of affected children [5,6,7,8,9,10,11]. Furthermore, it is associated with a high rate of healthcare utilization, contributing significantly to increased annual healthcare costs [12,13,14]. The Rome IV criteria classify IBS into different subtypes based on the stool consistency on days with abnormal bowel movements. These subtypes include IBS-C (constipation-predominant), IBS-D (diarrhea-predominant), IBS-M (mixed type) and IBS-U (unsubtyped) [7,15]. Patients with IBS-C have more than 25% of bowel movements classified as Bristol Stool Form Scale (BSFS) types 1 or 2, while patients with IBS-D have more than 25% of bowel movements classified as BSFS types 6 or 7. Patients with IBS-M experience more than 25% of bowel movements as types 1 or 2 and more than 25% as types 6 or 7 [16]. To date, the exact etiology of IBS remains unclear. Several mechanisms have been proposed, including increased intestinal permeability and gut dysbiosis [17,18]. For instance, crosstalk between the gut microbiota and enteroendocrine and enterochromaffin cells seems to stimulate neurotransmission between the intestinal epithelium and the nervous system, thereby influencing visceral pain through synaptic-like connections with local neural fibers [19,20]. As a result, the bidirectional brain–gut interactions—the so-called ‘‘brain–gut axis’’ and the more recent concept of “brain–gut-microbiota axis”—are key targets when considering pharmacological interventions in IBS [21]. There is strong evidence supporting the use of probiotics for improving overall symptoms in adults with IBS [22,23], and two meta-analyses have shown similar benefits in children with IBS [24,25]. However, these pediatric meta-analyses included data from randomized controlled trials (RCTs) investigating probiotics in children with IBS, as diagnosed using the Rome II criteria, as well as in children with functional abdominal pain. To provide updated and clinically relevant evidence, we conducted a systematic review and meta-analysis of randomized controlled trials (RCTs) evaluating the effects of probiotic supplementation on symptom improvement, specifically pain intensity, in pediatric patients diagnosed with IBS according to Rome III or IV criteria.

## 2. Materials and Methods

This systematic review was performed according to the Cochrane Handbook for Systematic Reviews and the Preferred Reporting Items for Systematic Reviews and Meta-Analyses [26]. The review protocol was registered at PROSPERO (identifier RD42025626829). Since this study did not involve primary data collection, the protocol was not submitted for Institutional Review Board approval and did not require informed consent.

### 2.1. Search Strategy, Study Selection and Inclusion Criteria

A systematic search was conducted in the Scopus, PubMed, and Cochrane Library databases. The search strategy used Medical Subject Headings (MeSH) terms combined with the Boolean operators (“AND,” “OR”) as follows: ((pro-biotics) OR (probiotics) OR (probiotic)) AND ((irritable bowel syndrome) OR (IBS)) AND ((randomized controlled trial) OR (RCT)) AND (children). No restriction was applied. The search was supplemented by scanning the reference lists of the retrieved articles. Duplicate articles were removed, and the title and abstract of all retrieved records were screened. Studies that did not meet the inclusion criteria were excluded from the analysis. Two researchers and reasons examined the full texts of potentially relevant articles for exclusion, and these were recorded. Any disagreements were resolved through discussion with a third author. The search spanned the period from 1 January 2010, to 30 September 2024, to exclude RCTs that enrolled children diagnosed with IBS according to the Rome I or Rome II criteria. Studies meeting the following criteria were selected: (1) randomized controlled trial design; (2) probiotics supplementation as the intervention; (3) participants were children and adolescents (under or equal to 18 years of age) with IBS, according to Rome III or IV criteria; and (4) sufficient data reported on abdominal pain severity scores. Exclusion criteria included: studies without control groups, non-randomized trials, studies conducted exclusively in adults, animal studies, studies without available full text, and non-English publications. Studies that did not report sufficient data on the predefined outcomes or that enrolled healthy subjects were also excluded.

### 2.2. Review Question

The review question was formulated according to the PICO framework (P: Population, I: Intervention, C: Comparison, O: Outcomes) as follows: in children with Irritable Bowel Syndrome (P), does probiotic treatment (I), compared to placebo (C), demonstrate superior efficacy in reducing abdominal pain as primary outcome (O) and in improving stool consistency and quality of life (QoL) scores, while also evaluating trial-related adverse events as secondary outcomes?

### 2.3. Data Extraction

A detailed full-text review was independently performed by two authors (G.Ga. and G.Gia.). The following data were abstracted using standardized pre-piloted forms: first author’s name, year of publication, study location, sample size, RCT design, type of probiotic supplement dose and duration of intervention, participants’ characteristics (gender, mean age), and the means and standard deviations (SDs) of outcome measures in both the intervention and control groups.

### 2.4. Quality Assessment

The methodological quality of eligible RCTs was assessed with the revised Risk of Bias tool for randomized trials (RoB2 tool) [27]. Each study was independently rated by two authors as high, low, or unclear risk of bias based on the following domains: allocation concealment, blinding of outcome assessment, blinding of participants and personnel, incomplete outcome data, random sequence generation, selective reporting, and other biases. Any disagreements were resolved through consultation with a third reviewer (M.P.).

### 2.5. Statistical Analysis

The effect of probiotics on pain intensity was considered the primary outcome. Outcomes were reported in terms of median, mean, standard deviation (SD) and confidence intervals (CIs) when available. For this meta-analysis, the primary data extracted included the mean and SD of pain intensity scores at baseline (pre-treatment) and after intervention (post-treatment) for both probiotic and placebo groups. In addition, the change in pain intensity scores from baseline to post-treatment was calculated separately for each group. Given the variability in pain assessment instruments between studies, results were harmonized by calculating standardized mean differences (SMDs) with corresponding 95% confidence intervals (CIs). A random-effects meta-analysis was conducted using the restricted maximum likelihood (REML) method. Statistical heterogeneity between studies was assessed using the I^2^ statistic, with values below 50% indicating low heterogeneity, 50–75% indicating moderate heterogeneity, and values above 75% indicating high heterogeneity. All analyses were performed using STATA (StataCorp LLC, 4905 Lakeway Drive, College Station, TX, USA), version 18.0.

## 3. Results

### 3.1. Study Selection

**Figure 1:**
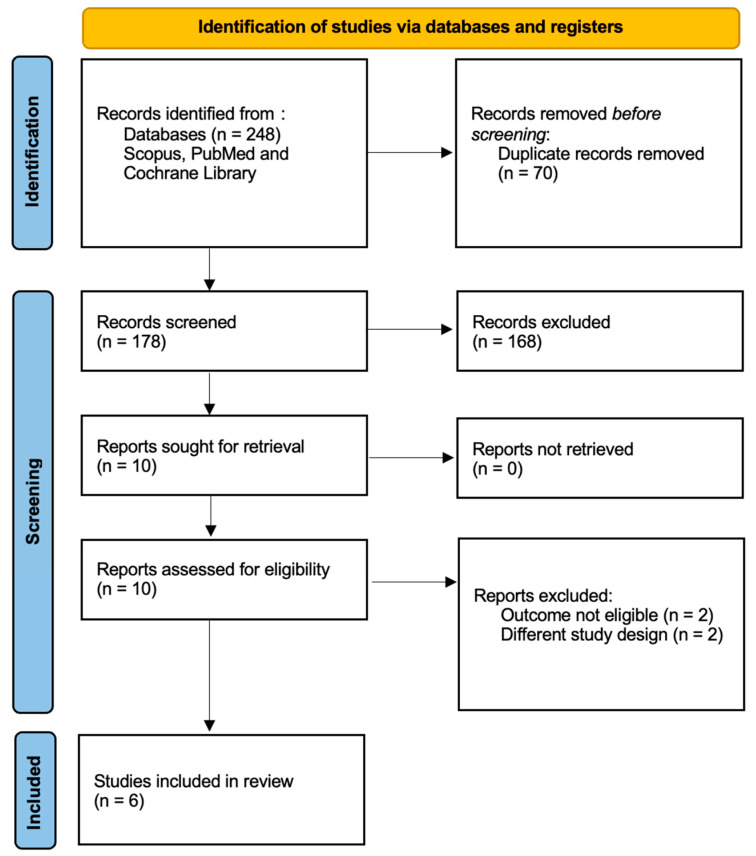
PRISMA flowchart of the study selection process.

Using our search criteria, a comprehensive electronic database search using PubMed, Scopus, and the Cochrane Library yielded a total of 248 records. After removing 70 duplicates, 178 articles were screened based on their titles and abstracts. Of these, 168 were excluded primarily because they were review articles (*n* = 107), involved adult populations (*n* = 19), were meta-analyses (*n* = 10), or lacked full-text availability (*n* = 10). Additional exclusions included publication types that did not meet the inclusion criteria (e.g., conference proceedings, commentaries), irrelevant outcomes, or non-probiotic interventions. Full-text review of the remaining ten studies resulted in the exclusion of four studies due to unsuitable design or outcomes. Ultimately, six randomized controlled trials (RCTs) were included in the systematic review and meta-analysis [28,29,30,31,32,33]. The study selection process is detailed in the PRISMA flow diagram (Figure 1).

### 3.2. Studies and Patient Characteristics

The 6 included RCTs involved a total of 604 pediatric patients (aged 4–18 years) diagnosed with irritable bowel syndrome (IBS) according to the Rome III or IV criteria (Supplementary Table S1). Among participants with reported sex (*n* = 595), females comprised 51.3% (*n* = 305) and males 48.7% (*n* = 290). All studies employed placebo-controlled designs, with intervention durations ranging from 4 to 12 weeks. Probiotic regimens varied, including single strains such as *Lactobacillus rhamnosus* GG [28], *Bacillus coagulans Unique* IS2 [29], *Bacillus clausii* [30], *Bifidobacterium adolescentis* PRL2019 [31], *Lactobacillus reuteri* [32], as well as multi-strain formulations containing *Bifidobacterium longum* BB536, *Bifidobacterium infantis* M-63, *Bifidobacterium breve* M16V [33]. Primary outcomes assessed across studies included the frequency and intensity of abdominal pain. Secondary outcomes included global symptom relief, stool consistency, and quality of life (QoL).

#### 3.2.1. Efficacy of Probiotics on Abdominal Pain Scores

All six RCTs evaluated probiotics’ effects on abdominal pain in pediatric IBS. Rahmani et al. found that 40% (6/15) of patients receiving probiotics experienced significant pain improvement versus none in placebo (*p* = 0.01) [32]. Kianifar et al. reported significant reductions in pain severity after four weeks of *Lactobacillus* GG (*p* < 0.01) [28]. Sudha et al. observed significant pain intensity decreases with *B. coagulans Unique* IS2 (*p* < 0.0001) [29]. Two studies documented higher rates of complete abdominal pain resolution in probiotic versus placebo groups (*p* = 0.003 and *p* = 0.006) [31,33]. Conversely, Vázquez-Frias et al. found no significant difference between *B. clausii* and placebo (*p* > 0.05) [30].

#### 3.2.2. Efficacy on Abdominal Distension and Bloating

Several studies assessed probiotics’ impact on abdominal distension and bloating. Sudha et al. observed reductions in abdominal discomfort and bloating with *B. coagulans* (*p* < 0.05) [29]. No significant differences were noted in the *B. clausii* study by Vázquez-Frias et al. (*p* > 0.05) [30].

#### 3.2.3. Effects on Bowel Habits

Probiotic-related improvements in bowel habits were reported by several trials. Sudha et al. reported better stool consistency, reduced incomplete evacuation, and urgency with *B. coagulans* (*p* < 0.05) [29]. Also Giorgio et al. reported better stool consistency in patients receiving *B.adolescentis* PRL2019 after treatment (*p* = 0.04) [31]. However, Kianifar et al. and Vázquez-Frias et al. found no significant differences compared to placebo [28,30].

#### 3.2.4. Quality of Life Outcomes

Quality of life was assessed in a limited subset of studies. Two studies reported significantly greater QoL improvement with probiotics versus placebo [31,33]. Other studies, including Vázquez-Frias et al., observed no significant differences [30].

### 3.3. Meta-Analysis

**Figure 2:**
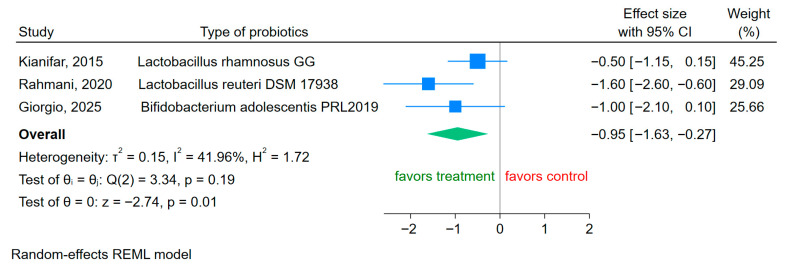
Forest plot of RCTs of the effects of probiotics (treatment) vs. placebo on abdominal pain in children with IBS [28,31,32]. Negative SMD values indicate a greater reduction in pain in the probiotic group (“favors treatment”). Effect sizes = standardized mean differences (SMDs) CI = 95% confidence intervals.

Data on the effect of probiotics on pain outcomes were reported in only three RCTs [28,31,32]. Probiotic supplementation resulted in a significant reduction in pain intensity compared to placebo (SMD −0.95, 95% CI −1.63 to −0.27; I^2^ = 41.96%) (Figure 2). Lower pain scores reflect symptom improvement, and therefore a negative effect size demonstrates the beneficial effect of probiotics.

**Figure 3:**
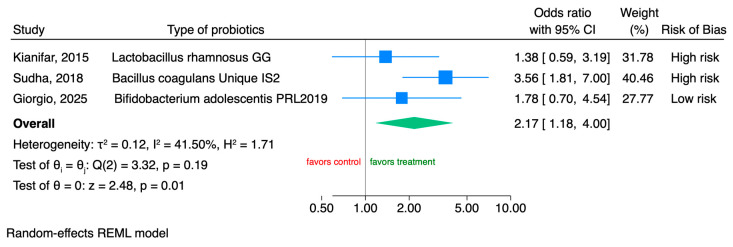
Forest plot of RCTs of the effects of probiotics (treatment) vs. placebo on stool consistency in children with IBS [28,29,31].

Only three RCTs [28,29,31] reported data on the effect of probiotics on stool consistency, according to the Bristol Stool Form Scale (BSFS). Probiotic supplementation resulted in significant normalization of bowel habits in IBS patients with diarrhea or constipation at baseline compared to placebo (OR 2.17, 95% CI 1.18 to 4, I^2^ = 41.50%) (Figure 3).

## 4. Discussion

This updated systematic review and meta-analysis provide compelling evidence for the beneficial effects of probiotics in treating IBS in pediatric patients.

Probiotics are live, nonpathogenic microorganisms known to exert several beneficial effects, including modulation of the host immune response within the gastrointestinal tract and suppression of pathogenic bacterial growth by promoting microbial balance [34]. Although IBS pathogenesis is multifaceted and not yet entirely understood, it is currently recognized as a brain–gut disorder. In IBS patients, a dysregulation between the enteric and central nervous systems occurs, resulting in altered sensations, motility, and potentially immune dysfunction [35]. In this scenario, gut microbiota plays a pivotal role, leading to the updated concept of the “brain–gut-microbiome axis,” which has replaced the earlier “brain–gut axis” model [36]. Probiotics can potentially modulate multiple components of this axis simultaneously, including restoring microbial balance, producing neuroactive compounds, reducing intestinal inflammation, and strengthening epithelial barrier function. Consequently, current guidelines support the use of probiotic supplementation in both adults and children with IBS [37,38]. Several studies have reported a positive correlation between IBS severity and low microbial diversity. In particular, reduced alpha and beta diversity of the gut have been associated with impaired intestinal motility and altered stool consistency [39,40]. Some adult studies have linked IBS severity to the absence of Methanobacteriales and the reduced presence of Bacteroides enterotypes [41]. Additionally, Pozuelo and colleagues observed a lower abundance of butyrate-producing and methane-producing bacteria in adult IBS-D and IBS-M patients [42]. In our analysis, probiotic supplementation led to a significant reduction in abdominal pain compared to the placebo. Similar to our findings, two previous meta-analyses have also demonstrated that probiotic supplementation reduces abdominal pain in children with IBS [24,25]. Experimental studies in germ-free animals have shown that dysbiosis can alter the perception of inflammatory visceral pain, highlighting the importance of the microbiota in regulating gut motility and sensory signaling [43]. Additionally, a disrupted gut barrier (“leaky gut”) can facilitate the translocation of luminal antigens, triggering immune responses and the release of pro-inflammatory mediators—such as histamine, tryptase, serotonin, and several polyunsaturated fatty acid derivatives (e.g., 12-HETE, 15-HETE, 5-HETE, 5-oxo-ETE, and LTB4)—all of which may contribute to sensory afferent hypersensitivity and abdominal pain [44,45,46]. Emerging evidence also supports the association between IBS and the reduced stability and biodiversity of the gut microbiota [18]. For instance, an Italian cross-sectional adult study revealed significant differences in Clostridiales abundance across IBS subtypes, which were correlated with fecal short-chain fatty acid (SCFA) levels and cytokine profiles [47]. Numerous studies suggested that probiotics may have an anti-inflammatory effect. For example, improvement in symptoms with Bifidobacteria supplementation has been demonstrated to be associated with an increased production of anti-inflammatory cytokines (IL-12) [48], and *L. reuteri* in an experimental rodent study reduced tumor necrosis factor-alpha (TNF-alpha)-induced production of IL-8 [49,50].

The present meta-analysis also demonstrated the beneficial effects of probiotic supplementation on stool consistency, helping to restore normal intestinal transit at the end of treatment in children with either constipation or diarrhea. Reduced ileal retention (and therefore increased colonic delivery) of bile acids has been suggested as a potential cause for symptoms in patients with functional diarrhea [51]. Lactobacilli and Bifidobacteria subspecies can deconjugate and absorb bile acids. Thus, one presumptive mechanism of probiotics may be rooted in their ability to reduce bile salt load to the colon, thereby avoiding potential colonic secretion and mucosal permeability changes induced by salt [50]. Gut microbiota commonly metabolize dietary substrates, producing gas and short-chain fatty acids. The latter may induce propulsive contractions, accelerate transit, or enhance fluid and sodium absorption in the colon. Methane-producing bacteria predominate in patients with IBS-C, and the amount of gas released is directly correlated with the severity of constipation [52]. In addition, in these patients, the presence of these bacteria is associated with slower passage through the intestine, reduced segmental contraction, and attenuated propulsion [53] Thus, probiotics supplementation may modify the colonic metabolism, influencing colonic transit and stool consistency [50].

Despite these promising findings, a key limitation must be acknowledged: the vast heterogeneity of probiotic strains, doses, and treatment durations included in the existing literature. While our meta-analysis shows a significant overall effect, the results cannot be generalized to all probiotic formulations. Emerging evidence highlights that the clinical efficacy of probiotics is highly strain-specific, emphasizing that our findings represent the general effect of probiotics rather than strain-specific recommendations, and that caution is warranted when generalizing results to specific probiotic products. A large meta-analysis by McFarland et al., which included both adult and pediatric IBS populations, demonstrated that certain individual strains—such as *B. coagulans* MTCC5260, *Lactobacillus plantarum* 299v, *Saccharomyces boulardii* CNCM I-745, and *Saccharomyces cerevisiae* CNCM I-3856—were significantly more effective than placebo in improving specific IBS symptoms, particularly abdominal pain [54]. However, even in that extensive analysis, the considerable variability in study design, outcome definitions, and probiotic formulations limited the ability to recommend a universal strain or formulation. Moreover, in our review, the six included RCTs tested six different strains or combinations, ranging from spore-forming probiotics (e.g., *B.* *clausii*) to traditional lactic acid bacteria (e.g., *L.* *rhamnosus* GG, *B. adolescentis* PRL2019). The diversity in formulation and strain characteristics—including their resistance to gastric acid, adhesion capacity, and immunomodulatory effects—likely contributes to the variability in clinical outcomes. Additionally, the gut microbiota profile in pediatric IBS patients lacks a clearly defined or characteristic signature and may have distinct microbiome characteristics compared to adult disease due to developmental maturation of the gut microbiota during childhood and adolescence, further complicating the identification of targeted probiotic interventions. Therefore, while probiotics appear to be a promising adjunctive treatment, personalized approaches based on strain functionality and patient microbiota profiling may be needed to optimize outcomes. Current guidelines, including the European Society for Paediatric Gastroenterology Hepatology and Nutrition (ESPGHAN) position paper [55], acknowledge these limitations and emphasize the need for caution when interpreting results from probiotic studies that use broad or heterogeneous definitions. They advocate for a more targeted approach—highlighting the importance of selecting specific strains, optimizing treatment duration and dosing, and designing trials that account for patient heterogeneity. Notably, ESPGHAN goes beyond a generic recommendation and, based on the currently available high-quality evidence, identifies two strains with sufficient efficacy and safety data in children with functional gastrointestinal disorders: *L. reuteri* DSM 17938 and *L. rhamnosus* GG. This point is particularly relevant, as it highlights how the field is shifting from the broad and non-specific use of probiotics toward evidence-based, strain-specific guidance. Nevertheless, even for these two strains, the recommendations are condition-specific (e.g., functional abdominal pain and colic), and their role in pediatric IBS still requires further validation [55].

## 5. Conclusions

In conclusion, this updated systematic review and meta-analysis suggest that probiotic supplementation may offer clinical benefits in alleviating symptoms of irritable bowel syndrome in children, particularly in reducing abdominal pain and improving stool consistency. Considering the complexity of the analyzed variables, the interpretation of these findings requires careful consideration and caution. The significant heterogeneity in probiotic strains and treatment protocols across included studies prevents the formulation of strain-specific clinical recommendations, nor can the results be generalized to a specific genus or species. As documented in prior literature, probiotic efficacy appears to be both strain-dependent and outcome-specific, and not all formulations yield the same benefits. Additionally, the limited number of high-quality pediatric trials and variability in outcome measures further restrict the generalizability of our findings.

Future research should aim to identify the most effective probiotic strains for each IBS subtype, using standardized diagnostic criteria and clinically meaningful endpoints. The integration of microbiota profiling and biomarkers may also support a move toward precision probiotic therapy in pediatric IBS.

## Data Availability

No new data were created or analyzed in this study. Data sharing is not applicable to this article.

## Supplementary Materials

The following supporting information can be downloaded at: https://www.mdpi.com/article/10.3390/microorganisms14010023/s1, Table S1: Characteristics of Randomized Controlled Trials of Probiotics Versus Placebo in Children with Irritable Bowel Syndrome.

## Abbreviations

The following abbreviations are used in this manuscript:

IBS	Irritable Bowel Syndrome
DGBI	Disorder of Gut–brain Interaction
IBS-C	Irritable Bowel Syndrome constipation-predominant
IBS-D	Irritable Bowel Syndrome diarrhea-predominant
IBS-M	Irritable Bowel Syndrome mixed type
IBS-U	Irritable Bowel Syndrome-unsubtyped
BSFS	Bristol Stool Form Scale
RCT	Randomized Controlled Trial
MESH	Medical Subject Headings
PICO	Population, Intervention, Comparison, Outcomes
QoL	Quality of Life
SD	Standard Deviation
CI	Confidence Interval
SMD	Standardized Mean Difference
SEs	Standard errors
REML	Restricted Maximum Likelihood
ESPGHAN	European Society for Paediatric Gastroenterology Hepatology and Nutrition

## References

[1] Hyams J.S., Di Lorenzo C., Saps M., Shulman R.J., Staiano A., van Tilburg M. (2016). Functional Disorders: Children and Adolescents. Gastroenterology.

[2] Devanarayana N.M., Rajindrajith S. (2018). Irritable bowel syndrome in children: Current knowledge, challenges and opportunities. World J. Gastroenterol..

[3] Liu D., Li D., Xu X., Lu H. (2005). An epidemiologic study of irritable bowel syndrome in adolescents and children in China: A school-based study. Pediatrics.

[4] Karabulut G.S., Beşer Ö.F., Erginöz E., Kutlu T., Çokuğraş F.Ç., Erkan T. (2013). The incidence of irritable bowel syndrome in children using the Rome III criteria and the effect of trimebutine treatment. J. Neurogastroenterol. Motil..

[5] Adeniyi O.F., Adenike Lesi O., Olatona F.A., Esezobor C.I., Ikobah J.M. (2017). Irritable bowel syndrome in adolescents in Lagos. Pan Afr. Med. J..

[6] Rajindrajith S., Devanarayana N.M. (2012). Subtypes and Symptomatology of Irritable Bowel Syndrome in Children and Adolescents: A School-based Survey Using Rome III Criteria. J. Neurogastroenterol. Motil..

[7] Giannetti E., de’Angelis G., Turco R., Campanozzi A., Pensabene L., Salvatore S., de Seta F., Staiano A. (2014). Subtypes of irritable bowel syndrome in children: Prevalence at diagnosis and at follow-up. J. Pediatr..

[8] Devanarayana N.M., Rajindrajith S., Benninga M.A. (2014). Quality of life and health care consultation in 13 to 18 year olds with abdominal pain predominant functional gastrointestinal diseases. BMC Gastroenterol..

[9] Ranasinghe N., Devanarayana N.M., Rajindrajith S., Perera M.S., Nishanthinie S., Warnakulasuriya T., de Zoysa P.T. (2018). Functional gastrointestinal diseases and psychological maladjustment, personality traits and quality of life. BMC Gastroenterol..

[10] Sagawa T., Okamura S., Kakizaki S., Zhang Y., Morita K., Mori M. (2013). Functional gastrointestinal disorders in adolescents and quality of school life. J. Gastroenterol. Hepatol..

[11] Varni J.W., Lane M.M., Burwinkle T.M., Fontaine E.N., Youssef N.N., Schwimmer J.B., Pardee P.E., Pohl J.F., Easley D.J. (2006). Health-related quality of life in pediatric patients with irritable bowel syndrome: A comparative analysis. J. Dev. Behav. Pediatr..

[12] Park R., Mikami S., LeClair J., Bollom A., Lembo C., Sethi S., Lembo A., Jones M., Cheng V., Friedlander E. (2015). Inpatient burden of childhood functional GI disorders in the USA: An analysis of national trends in the USA from 1997 to 2009. Neurogastroenterol. Motil..

[13] Hoekman D.R., Rutten J.M., Vlieger A.M., Benninga M.A., Dijkgraaf M.G. (2015). Annual Costs of Care for Pediatric Irritable Bowel Syndrome, Functional Abdominal Pain, and Functional Abdominal Pain Syndrome. J. Pediatr..

[14] Lane M.M., Weidler E.M., Czyzewski D.I., Shulman R.J. (2009). Pain symptoms and stooling patterns do not drive diagnostic costs for children with functional abdominal pain and irritable bowel syndrome in primary or tertiary care. Pediatrics.

[15] Self M.M., Czyzewski D.I., Chumpitazi B.P., Weidler E.M., Shulman R.J. (2014). Subtypes of irritable bowel syndrome in children and adolescents. Clin. Gastroenterol. Hepatol..

[16] Lacy B.E., Patel N.K. (2017). Rome Criteria and a Diagnostic Approach to Irritable Bowel Syndrome. J. Clin. Med..

[17] Ford A.C., Lacy B.E., Talley N.J. (2017). Irritable Bowel Syndrome. N. Engl. J. Med..

[18] Durbán A., Abellán J.J., Jiménez-Hernández N., Artacho A., Garrigues V., Ortiz V., Ponce J., Latorre A., Moya A. (2013). Instability of the faecal microbiota in diarrhoea-predominant irritable bowel syndrome. FEMS Microbiol. Ecol..

[19] Najjar S.A., Albers K.M. (2021). Pain in Inflammatory Bowel Disease: Optogenetic Strategies for Study of Neural-Epithelial Signaling. Crohns Colitis.

[20] El-Salhy M., Hausken T., Gilja O.H., Hatlebakk J.G. (2017). The possible role of gastrointestinal endocrine cells in the pathophysiology of irritable bowel syndrome. Expert Rev. Gastroenterol. Hepatol..

[21] Sandhu B.K., Paul S.P. (2014). Irritable bowel syndrome in children: Pathogenesis, diagnosis and evidence-based treatment. World J. Gastroenterol..

[22] Ding L., Ning L., Xu G. (2019). Efficacy of different probiotic protocols in irritable bowel syndrome: A network meta-analysis. Medicine.

[23] Goodoory V.C., Khasawneh M., Black C.J., Quigley E.M.M., Moayyedi P., Ford A.C. (2023). Efficacy of Probiotics in Irritable Bowel Syndrome: Systematic Review and Meta-analysis. Gastroenterology.

[24] Xu H.L., Zou L.L., Chen M.B., Wang H., Shen W.M., Zheng Q.H., Cui W.Y. (2021). Efficacy of probiotic adjuvant therapy for irritable bowel syndrome in children: A systematic review and meta-analysis. PLoS ONE.

[25] Fatahi S., Hosseini A., Sohouli M.H., Sayyari A., Khatami K., Farsani Z.F., Amiri H., Dara N., de Souza I.G.O., Santos H.O. (2022). Effects of probiotic supplementation on abdominal pain severity in pediatric patients with irritable bowel syndrome: A systematic review and meta-analysis of randomized clinical trials. World J. Pediatr..

[26] Higgins J.P.T., Thomas J., Chandler J., Cumpston M., Li T., Page M.J. (2024). Cochrane Handbook for Systematic Reviews of Interventions Version 6.5.

[27] Sterne J.A.C., Savović J., Page M.J., Elbers R.G., Blencowe N.S., Boutron I., Cates C.J., Cheng H.Y., Corbett M.S., Eldridge S.M. (2019). RoB 2: A revised tool for assessing risk of bias in randomised trials. BMJ.

[28] Kianifar H., Jafari S.A., Kiani M., Ahanchian H., Ghasemi S.V., Grover Z., Mahmoodi L.Z., Bagherian R., Khalesi M. (2015). Probiotic for irritable bowel syndrome in pediatric patients: A randomized controlled clinical trial. Electron. Physician.

[29] Sudha M.R., Jayanthi N., Aasin M., Dhanashri R.D., Anirudh T. (2018). Efficacy of Bacillus coagulans Unique IS2 in treatment of irritable bowel syndrome in children: A double blind, randomised placebo controlled study. Benef. Microbes.

[30] Vázquez-Frias R., Consuelo-Sánchez A., Acosta-Rodríguez-Bueno C.P., Blanco-Montero A., Robles D.C., Cohen V., Márquez D., Perez M. (2023). Efficacy and Safety of the Adjuvant Use of Probiotic Bacillus clausii Strains in Pediatric Irritable Bowel Syndrome: A Randomized, Double-Blind, Placebo-Controlled Study. Paediatr. Drugs.

[31] Giorgio V., Quatrale G., Mennini M., Piccirillo M., Furio S., Stella G., Ferretti A., Parisi P., Evangelisti M., Felici E. (2025). Bifidobacterium adolescentis PRL2019 in Pediatric Irritable Bowel Syndrome: A Multicentric, Randomized, Double-Blind, Placebo-Controlled Trial. Microorganisms.

[32] Rahmani P., Ghouran-Orimi A., Motamed F., Moradzadeh A. (2020). Evaluating the effects of probiotics in pediatrics with recurrent abdominal pain. Clin. Exp. Pediatr..

[33] Giannetti E., Maglione M., Alessandrella A., Strisciuglio C., De Giovanni D., Campanozzi A., Miele E., Staiano A. (2017). A Mixture of 3 Bifidobacteria Decreases Abdominal Pain and Improves the Quality of Life in Children with Irritable Bowel Syndrome: A Multicenter, Randomized, Double-Blind, Placebo-Controlled, Crossover Trial. J. Clin. Gastroenterol..

[34] Williams N. (2010). Probiotics. Am. J. Health-Syst. Pharm..

[35] Camilleri M. (2012). Peripheral mechanisms in irritable bowel syndrome. N. Engl. J. Med..

[36] Martin C.R., Osadchiy V., Kalani A., Mayer E.A. (2018). The Brain-Gut-Microbiome Axis. Cell. Mol. Gastroenterol. Hepatol..

[37] Di Nardo G., Barbara G., Borrelli O., Cremon C., Giorgio V., Greco L., La Pietra M., Marasco G., Pensabene L., Piccirillo M. (2024). Italian guidelines for the management of irritable bowel syndrome in children and adolescents: Joint Consensus from the Italian Societies of: Gastroenterology, Hepatology and Pediatric Nutrition (SIGENP), Pediatrics (SIP), Gastroenterology and Endoscopy (SIGE) and Neurogastroenterology and Motility (SINGEM). Ital. J. Pediatr..

[38] Barbara G., Cremon C., Bellini M., Corsetti M., Di Nardo G., Falangone F., Fuccio L., Galeazzi F., Iovino P., Sarnelli G. (2023). Italian guidelines for the management of irritable bowel syndrome: Joint Consensus from the Italian Societies of: Gastroenterology and Endoscopy (SIGE), Neurogastroenterology and Motility (SINGEM), Hospital Gastroenterologists and Endoscopists (AIGO), Digestive Endoscopy (SIED), General Medicine (SIMG), Gastroenterology, Hepatology and Pediatric Nutrition (SIGENP) and Pediatrics (SIP). Dig. Liver Dis..

[39] Roager H.M., Hansen L.B., Bahl M.I., Frandsen H.L., Carvalho V., Gøbel R.J., Dalgaard M.D., Plichta D.R., Sparholt M.H., Vestergaard H. (2016). Colonic transit time is related to bacterial metabolism and mucosal turnover in the gut. Nat. Microbiol..

[40] Benjak Horvat I., Gobin I., Kresović A., Hauser G. (2021). How can probiotic improve irritable bowel syndrome symptoms?. World J. Gastrointest. Surg..

[41] Tap J., Derrien M., Törnblom H., Brazeilles R., Cools-Portier S., Doré J., Störsrud S., Le Nevé B., Öhman L., Simrén M. (2017). Identification of an Intestinal Microbiota Signature Associated with Severity of Irritable Bowel Syndrome. Gastroenterology.

[42] Pozuelo M., Panda S., Santiago A., Mendez S., Accarino A., Santos J., Guarner F., Azpiroz F., Manichanh C. (2015). Reduction of butyrate- and methane-producing microorganisms in patients with Irritable Bowel Syndrome. Sci. Rep..

[43] Amaral F.A., Sachs D., Costa V.V., Fagundes C.T., Cisalpino D., Cunha T.M., Ferreira S.H., Cunha F.Q., Silva T.A., Nicoli J.R. (2008). Commensal microbiota is fundamental for the development of inflammatory pain. Proc. Natl. Acad. Sci. USA.

[44] Cenac N., Bautzova T., Le Faouder P., Veldhuis N.A., Poole D.P., Rolland C., Bertrand J., Liedtke W., Dubourdeau M., Bertrand-Michel J. (2015). Quantification and Potential Functions of Endogenous Agonists of Transient Receptor Potential Channels in Patients with Irritable Bowel Syndrome. Gastroenterology.

[45] Bautzova T., Hockley J.R.F., Perez-Berezo T., Pujo J., Tranter M.M., Desormeaux C., Barbaro M.R., Basso L., Le Faouder P., Rolland C. (2018). 5-oxoETE triggers nociception in constipation-predominant irritable bowel syndrome through MAS-related G protein-coupled receptor D. Sci. Signal..

[46] Barbara G., Wang B., Stanghellini V., de Giorgio R., Cremon C., Di Nardo G., Trevisani M., Campi B., Geppetti P., Tonini M. (2007). Mast cell-dependent excitation of visceral-nociceptive sensory neurons in irritable bowel syndrome. Gastroenterology.

[47] Gargari G., Taverniti V., Gardana C., Cremon C., Canducci F., Pagano I., Barbaro M.R., Bellacosa L., Castellazzi A.M., Valsecchi C. (2018). Fecal Clostridiales distribution and short-chain fatty acids reflect bowel habits in irritable bowel syndrome. Environ. Microbiol..

[48] O’Mahony L., McCarthy J., Kelly P., Hurley G., Luo F., Chen K., O’Sullivan G.C., Kiely B., Collins J.K., Shanahan F. (2005). Lactobacillus and bifidobacterium in irritable bowel syndrome: Symptom responses and relationship to cytokine profiles. Gastroenterology.

[49] Ma D., Forsythe P., Bienenstock J. (2004). Live *Lactobacillus rhamnosus* [corrected] is essential for the inhibitory effect on tumor necrosis factor alpha-induced interleukin-8 expression. Infect. Immun..

[50] Camilleri M. (2006). Probiotics and irritable bowel syndrome: Rationale, putative mechanisms, and evidence of clinical efficacy. J. Clin. Gastroenterol..

[51] Brydon W.G., Nyhlin H., Eastwood M.A., Merrick M.V. (1996). Serum 7 alpha-hydroxy-4-cholesten-3-one and selenohomocholyltaurine (SeHCAT) whole body retention in the assessment of bile acid induced diarrhoea. Eur. J. Gastroenterol. Hepatol..

[52] Chatterjee S., Park S., Low K., Kong Y., Pimentel M. (2007). The degree of breath methane production in IBS correlates with the severity of constipation. Am. J. Gastroenterol..

[53] Agrawal A., Houghton L.A., Morris J., Reilly B., Guyonnet D., Goupil Feuillerat N., Schlumberger A., Jakob S., Whorwell P.J. (2009). Clinical trial: The effects of a fermented milk product containing Bifidobacterium lactis DN-173 010 on abdominal distension and gastrointestinal transit in irritable bowel syndrome with constipation. Aliment. Pharmacol. Ther..

[54] McFarland L.V., Karakan T., Karatas A. (2021). Strain-specific and outcome-specific efficacy of probiotics for the treatment of irritable bowel syndrome: A systematic review and meta-analysis. eClinicalMedicine.

[55] Szajewska H., Canani R.B., Domellöf M., Guarino A., Hojsak I., Indrio F., Vecchio A.L., Mihatsch W.A., Mosca A., Salvatore S. (2023). Probiotics for the management of pediatric gastrointestinal disorders: Position paper of the ESPGHAN Special Interest Group on Gut Microbiota and Modifications. J. Pediatr. Gastroenterol. Nutr..

