# Supplementary material for: Efficacy and Safety of Probiotics in Children with Irritable Bowel Syndrome: A Systematic Review and Meta-Analysis
Source: Microorganisms. 2025 Dec 21;14(1):23. doi: 10.3390/microorganisms14010023 (PMC12844155; doi:10.3390/microorganisms14010023)
Supplement: Supplementary file 1 [file microorganisms-14-00023-s001.zip › microorganisms-4026173-supplementary.pdf]

Supplementary Table S1: Characteristics of Randomized Controlled Trials of Probiotics Versus Placebo in Children with Irritable Bowel Syndrome

Author, year	*Study design*	*Country*	*Sample IBS (N, age, sex)*	*Primary Outcome*	*Criteria for diagnosis*	*Treatment follow-up*	*Measurements*	*Treatment*	*Control*	*Quality assessment*
Giannetti, 2017	*Multicenter, randomized, double-blind, placebo-controlled, crossover trial*	*Italy*	*48 participants<br><br>Mean age, 11.2 y; range, 8 to 17.9 y<br><br>female:male 21:27*	*Efficacy in reducing gastrointestinal symptoms (abdominal pain resolution)*	*Rome III criteria*	*6 weeks*	*Questionnaire on Pediatric Gastrointestinal Symptoms, Rome III Version (QPGS-RIII)<br><br>Functional Disability Inventory" (FDI)*	*B. longum BB536 + B.infantis M-63 + B. breve M-16V*	*Placebo*	*High risk*
Kianifar, 2015	*Double-blind, randomized controlled trial*	*Iran*	*60 participants<br><br>Mean age 7.1 ± 0.3 years<br><br>female:male 25:27*	*Efficacy in reducing gastrointestinal symptoms (pain severity)*	*Rome III criteria*	*4 weeks*	*Pain Severity Scale*	*Lactobacillus rhamnosus GG*	*Placebo*	*High risk*
Rahmani,	*Double-blind, randomized*	*Iran*	*30*	*Efficacy in reducing gastrointestinal*	*Rome III*	*4 weeks*	*Wang-Baker FACES Pain Rating*	*Lactobacillus*	*Placebo*	*High risk*

2020	*controlled trial*		*participants*	*symptoms (pain severity)*	*criteria*		*Scale (WBFPRS) 2*	*reuteri*		
Sudha, 2018	*Double-blind, randomized controlled trial*	*India*	*141 participants*<br><br>*Mean age*<br>*7.9 years*<br>*range 4*<br>*to 12 y*<br><br>*female:male*<br>*61:80*	*Efficacy in reducing gastrointestinal symptoms (abdominal pain resolution)*	*Rome III criteria*	*8 weeks*	*Global Assessment of Relief for Children with IBS (SGARC)*<br>*Bristol stool scale*<br>*Pain Severity score*<br>*(auto-structured)*	*Bacillus coagulans Unique IS2*	*Placebo*	*High risk*
Vázquez-Frias, 2023	*Double-blind, randomized controlled trial*	*Mexico*	*253*<br>*participants*<br><br>*female:male*<br><br>*157:102*	*Efficacy and safety in reducing gastrointestinal symptoms (clinical improvement of symptoms)*	*Rome III criteria*	*8 weeks*	*Global Assessment of Relief for Children with IBS (SGARC)*<br><br>*Global*<br>*Assessment Questions (GAQ)*<br><br>*Bristol stool scale*<br><br>*Face*<br>*Pain Scale–*	*Bacillus clausii*	*Placebo*	*High risk*

							Revised<br><br>*IBS behavior (Behavior Scale).*			
Giorgio, 2025	*Double-blind, randomized controlled trial*	*Italy*	*72 participants*<br><br>*Median age 12.2 ± 1.8 years*<br><br>*female:male 42:30*	*Efficacy in reducing gastrointestinal symptoms (abdominal pain resolution)*	*Rome IV criteria*	*12 weeks*	*Total IBS-Symptom Severity Scale (IBS-SSS)*<br><br>*Bristol Stool Chart*	*Bifidobacterium adolescentis PRL2019*	*Placebo*	*Low risk*
